# Family-based analysis of eight susceptibility loci in polycystic ovary syndrome

**DOI:** 10.1038/srep12619

**Published:** 2015-07-29

**Authors:** Shigang Zhao, Ye Tian, Xuan Gao, Xiuqing Zhang, Hongbin Liu, Li You, Yongzhi Cao, Shizhen Su, Wai-Yee Chan, Yun Sun, Han Zhao, Zi-Jiang Chen

**Affiliations:** 1 Shanghai Key Laboratory for Assisted Reproduction and Reproductive Genetics, Center for Reproductive Medicine, Renji Hospital, School of Medicine, Shanghai Jiaotong University, Shanghai, China; 2 Center for Reproductive Medicine, Provincial Hospital Affiliated to Shandong University, Jinan, China; National Research Center for Assisted Reproductive Technology and Reproductive Genetics, China; The Key laboratory for Reproductive Endocrinology of Ministry of Education, China; Shandong Provincial Key Laboratory of Reproductive Medicine, Jinan, China; 3 The Chinese University of Hong Kong-Shandong University Joint Laboratory on Reproductive Genetics, School of Biomedical Sciences, The Chinese University of Hong Kong, Hong Kong SAR, China

## Abstract

Polycystic ovary syndrome (PCOS) is a complex endocrine disorder that is proposed to have a genetic basis. A recent genome-wide association study (GWAS) identified eight new risk loci that are independently associated with PCOS. To further validate the findings, a total of 321 case-parent trios (963 participants) who had a proband affected with PCOS were recruited for the family-based study. The transmission disequilibrium test (TDT) was used to analyze associations between PCOS and ten single nucleotide polymorphisms (SNPs) mapped to eight new susceptibility loci. Significant differences in transmission were observed for the SNPs rs2349415 (located in the *FSHR* gene, *P* = 0.0001) and rs3802457 (located in the *C9orf3* gene, *P* = 0.0001), even after correction for multiple testing bias. The present data provides further evidence for an association between two susceptibility loci, 2p16.3 and 9q22.32, and PCOS. Follow-up functional studies on the *FSHR* and *C9orf3* genes are required to understand their roles in PCOS development.

Polycystic ovary syndrome (PCOS) is a complex, heterogeneous disorder characterized by chronic anovulation, clinical and/or biochemical hyperandrogenism and polycystic ovaries^1^. It is the most common endocrine disorder in women of reproductive age, with a prevalence of 5%–10%^2^. Associated symptoms of PCOS include obesity, insulin resistance, type 2 diabetes and cardiovascular disease, which can lead to major health issues^3^. However, the etiology of PCOS is poorly understood.

PCOS is considered as a polygenic trait, and both genetic and environmental factors play important roles in the development of PCOS^4^. Studies from PCOS families and twins suggest heritability and a strong genetic basis^5^,^6^. Numerous candidate genes involved in the androgen biosynthetic pathway, insulin resistance and chronic inflammation have been identified as candidate genes for PCOS^7^. However, the mode of inheritance for PCOS has not been firmly established, and findings from candidate gene studies are inconsistent.

Recently, genome-wide association studies (GWAS) have facilitated detection of susceptibility genes in complex diseases. Our group conducted the first GWAS for PCOS (PCOS GWAS-I) and identified three susceptibility loci (2p16.3, 2p21 and 9q33.3) associated with PCOS in a Han Chinese population^8^. Candidate genes *LHCGR, THADA* and *DENND1A* mapped to these three regions, respectively. A subsequent GWAS (PCOS GWAS-II) was conducted using a larger sample size to cover all PCOS phenotypes^9^. Eight new loci were discovered and genes, including *FSHR, INSR, YAP1, HMGA2, C9orf3, RAB5B, TOX3* and *SUMO1P1,* were identified as candidates. A disadvantage of analyzing population-based GWAS data is difficulty ensuring that genetic differences between cases and controls are due solely to differences in disease status and not to differences in genetic background. Spurious associations can occur if there are population substructure or admixture^10^,^11^. Although cases and controls were matched carefully by geography in both of the PCOS GWAS-I and GWAS-II studies, it was difficult to classify Chinese samples due to the extremely long and complex demographic history of the Han Chinese. In addition to the principal component analysis (PCA) that was conducted in previous GWAS studies, another option is to perform case-parent trios analysis using the transmission disequilibrium test (TDT), which is robust to avoid population substructure and admixture.

The study of trios, including an affected proband and her parents, is the most basic family-based design for association testing. TDT is commonly used in trio studies. TDT recognizes distortions in transmission of alleles from parents to the affected proband^12^. Under no association with the disease, both alleles have an equal chance of being transmitted from a heterozygous parent (50% probability of transmission). If one allele is preferentially transmitted to the offspring affected with PCOS, an association between the polymorphisms and PCOS may exist. After the PCOS GWAS-I, TDT was conducted in 276 PCOS family trios to avoid the impact of underlying population stratification or genetic background differences in the case-control study^13^. The results demonstrated that the positive association between 2p16.3 (*THADA* gene) and PCOS is not likely attributable to population stratification and provided further support for 2p16.3 as a PCOS susceptibility loci. TDT is necessary to confirm associations between PCOS and eight new risk loci identified in the PCOS GWAS-II.

In the present study, ten SNPs from PCOS GWAS-II were investigated in 321 family trios with PCOS using TDT to provide further understanding regarding the relationship between eight new susceptibility loci and PCOS.

## Results

### Clinical and endocrine features

The mean age of PCOS cases was 27.06 ± 3.72 years and the BMI was 25.07 ± 4.53 kg/m^2^. Mean total testosterone was 69.04 ± 29.83 ng/dl, FSH level was 6.36 ± 1.73 IU/L and LH level was 11.27 ± 6.56 IU/L, as shown in Table 1.

### TDT analysis in trios with PCOS

For all SNPs evaluated, no Hardy Weinberg Equilibrium (HWE) deviations were detected, as shown in Table 2. Significant differences in transmission were found in rs2349415 (*FSHR* gene; transmitted trios: un-transmitted trios = 130:75, *P* = 0.0001), rs3802457 (*C9orf3* gene; transmitted trios: un-transmitted trios = 60:25, *P* = 0.0001) and rs2272046 (*HMGA2* gene; transmitted trios: un-transmitted trios = 50:31, *P* = 0.035). The marker rs4784165 had a mild transmission trend with PCOS (*TOX3* gene; transmitted trios: un-transmitted trios = 132:102, *P* = 0.050). And the remaining SNPs did not show significant associations with PCOS as shown in Table 2.

### Correction for multiple testing

To correct error generated by multiple calculations, we did permutation testing for association significance. After testing 10,000 permutations, only rs2349415 (*FSHR* gene, *P* = 0.0036) and rs3802457 (*C9orf3* gene, *P* = 0.0046) still showed significant differences.

## Discussion

To identify causative PCOS genes, our group previously conducted two genome-wide association studies (PCOS GWAS-I and PCOS GWAS-II) in Han Chinese women affected with PCOS and identified 11 susceptibility loci^8^,^9^. However, case-control studies are vulnerable to underlying population stratification, which may cause false-positive results. Hence, family-based analyses are necessary to avoid spurious associations due to population substructure and to further support GWAS results.

In this family-based study, we conducted TDT analysis for 10 significant SNPs identified in PCOS GWAS-II. Of the 10 SNPs, rs2349415 and rs3802457 demonstrated significant differences in transmission, even after correction for multiple testing. Previous PCOS GWAS data and results of this family-based TDT analysis indicate that 2p16.3 and 9q22.32 are associated with PCOS compared to false-positive data resulting from population structure.

The rs2349415 SNP is located in the intron region of the *FSHR* gene mapped to 2p16.3. The *FSHR* gene codes for the follicle stimulating hormone receptor, which is a member of the G protein-coupled receptor family and is expressed in the granulosa cells of the ovary^14^. Inherited abnormalities in *FSHR* expression could plausibly contribute to ovulatory dysfunction in PCOS. In women with PCOS undergoing controlled ovarian hyperstimulation, *FSHR* mRNA levels in granulosa cells from both small and large follicles were higher than in controls^15^. In previous studies, data regarding the association between *FSHR* and PCOS and two non-synonymous SNPs (rs6165 [Thr307Ala] and rs6166 [Ser680Asn]) in exon 10 are of particular interest. These two SNPs have been shown to be associated with PCOS in South Han Chinese^16^ and Korean (only rs6166)^17^ populations, but were not associated with PCOS in North Han Chinese populations^18^,^19^, nor the UK^20^,^21^, Netherlands^22^ and Singapore^23^, Bahraini Arab women^24^ and Turkish adolescent girls^25^. Although not associated with PCOS per se, *FSHR* Ser680 was found to be associated with higher levels of gonadotropic hormones and testosterone and a higher frequency of hyperandrogenism in women from the Netherlands affected with PCOS^26^. The mixed results may be due to distinct ethnic groups and the power of different studies. Our recent PCOS GWAS-I study included 65 *FSHR* SNPs and found that 13 SNPs had a PCA-adjusted *P* value ranging from 4.0E-04 to 2.0E-03, implying that the role of *FSHR* in PCOS could not be excluded^8^. The PCOS GWAS-II study identified two significantly associated SNPs in *FSHR*, rs2268361 and rs2349415 (*P*_meta_ = 9.89E-13, 2.35E-12, respectively), which indicated a strong association between *FSHR* and PCOS^9^. Furthermore, a replication study in a cohort of European ancestry provided evidence for an association between *FSHR* and PCOS, and the strongest SNP associations in the *FSHR* gene region were rs1922476 (*P* = 0.0053) and rs12994034 (*P* = 0.007)^27^,^28^. Meta-analysis of Chinese, US, and Dutch data demonstrated that SNPs in the *FSHR* region are significantly related to PCOS (rs2268361, *P*_meta_ = 3.8E-5 and rs2349415, *P*_meta_ = 3.6E-4), which indicates that *FSHR* is likely a common susceptibility gene for PCOS, regardless of ethnicity^29^. By extension, the functional effect of rs2349415 is predicted and indicated at the transcriptional regulation level by the F-SNP database^30^. Further studies are necessary to evaluate the exact effect of rs2349415 on *FSHR* expression.

SNP rs3802457 is located in the intron region of the *C9orf3* gene, which was mapped to 9q22.32. *C9orf3* has been shown to be a potential candidate for PCOS due to over-transmission of the risk allele C of rs3802457 in this family-based analysis. *C9orf3* encodes a zinc-dependent metallopeptidase, which catalyzes hydrolysis of amino acid residues from the N-terminus of peptides or protein substrates^31^. Previously, a GWAS on erectile dysfunction in a cohort of African-American prostate cancer patients found that two significant SNPs mapped to the *C9orf3* gene region (rs3802458 and rs10993429) with an unadjusted *P* value of approximately 4.0E-6^32^. The *C9orf3* gene was also suggested as a new atrial fibrillation susceptibility locus in individuals of European ancestry (rs10821415, *P*_meta_ = 4.2E-11)^33^. Cross-ethnic meta-analysis also demonstrated that rs3802457 was significantly correlated with PCOS (*P*_meta_ = 9.2E-6)^29^. Recently, our group found that *C9orf3* was related to all three PCOS features, which suggests that *C9orf3* may be involved in basic pathophysiological changes in PCOS^34^. Bioinformatic predictions show that C9orf3 interacts with ACVR1B (activin A receptor, type IB), INHBE (inhibin, beta E), ZP4 (zona pellucida glycoprotein 4) and others (see Supplementary Fig. S1 online)^35^. While ZP4 is solely expressed in oocytes, activin and inhibin belong to the TGF-β family, in which dysregulation may contribute to the pathogenesis of PCOS from several aspects^36^,^37^,^38^. These results call for future attention to research on the gene functions of *C9orf3,* which will benefit the understanding of PCOS etiologies.

Besides in PCOS, *FSHR* and *C9orf3* have both been identified as susceptibility genes for another reproductive disease—erectile dysfunction^32^. The result infers that both *FSHR* and *C9orf3* may have important roles in human reproduction. As known, C/EBPα and PI3K are pivotal effectors in folliculogenesis after FSH activating FSHR. Jue Feng *et al.* have identified that transcription factor C/EBPα could enhance transcription of endogenous *C9orf3* via activation of its promoter^39^. Additionally, McAllister *et al.* speculated that *C9orf3* might play roles through PI3K/MAPK signaling pathway^40^. *FSHR* and *C9orf3* may work together to mediate the pathogenesis of PCOS, which needs further studies to clarify.

The other 8 SNPs evaluated were not statistically associated with PCOS by TDT after corrections. The possibility that these gene variations could be related to PCOS cannot be excluded if we take several factors into consideration. First, PCOS is a complex disorder, which means that candidate genes are likely to control disease risk, and gene-gene or gene-environmental interactions may be important as well^4^. TDT analysis cannot account for environmental influences on PCOS development. Second, sample size is a limitation in this study. The present study included 321 PCOS trios, which is a relatively large sample size. However, only two SNPs (rs2349415 and rs3802457) reached 80% power with an α level of 0.05. For example, rs2272046 and rs4784165 would need 381 and 893 families to reach 80% power, respectively. In the future, we will recruit more families and select more SNPs for TDT analysis.

In summary, this family-based TDT analysis provides further evidence for an association between two susceptibility loci, 2p16.3 and 9q22.32, and PCOS. Follow-up functional studies on the *FSHR* and *C9orf3* genes are required to understand their roles in PCOS development.

## Methods

### PCOS families

The study evaluated 321 families consisting of women with PCOS and their first-degree relatives (father and mother). A total of 963 participants were recruited from the Center for Reproductive Medicine, Provincial Hospital Affiliated with Shandong University and the Center for Reproductive Medicine, Renji Hospital, School of Medicine, Shanghai Jiaotong University from July 2007 to February 2013. All PCOS probands were of Han Chinese origin and were not included in our previous GWAS. This study was approved by the institutional review boards of Shandong University and Shanghai Jiaotong University. Written informed consent was obtained from all participants. All methods were carried out in accordance with the approved guidelines.

All of the PCOS participants included in the study met the revised 2003 Rotterdam criteria with at least two of the following features: 1) oligomenorrhea or amenorrhea; 2) clinical or biochemical hyperandrogenism; or 3) polycystic ovaries on ultrasound; and exclusion of other related diseases, such as congenital adrenal hyperplasia, androgen-secreting tumors, Cushing’s syndrome, thyroid disease and hyperprolactinemia^1^.

Hyperandrogenism was defined on the basis of hirsutism (Ferriman-Gallwey score ≥6) or elevated circulating total testosterone ≥60 ng/dl. Polycystic ovaries were defined as the presence of at least 1 ovary >10 cm^3^ or containing at least 12 follicles 2–9 mm in diameter. Transvaginal ultrasound was used to detect polycystic ovaries, or ultrasound examination was performed rectally if the subjects were virginal.

### SNP genotyping

Genomic DNA was extracted from peripheral blood using a QIAamp DNA mini kit (QIAGEN, Hilden, Germany) according to the manufacturer’s protocol. All 10 SNPs (rs2268361, rs2349415, rs4385527, rs3802457, rs1894116, rs705702, rs2272046, rs4784165, rs2059807 and rs6022786) were genotyped with Sequenom MassArray (Beijing, China). A total of 5% of the samples were randomly selected for direct sequencing to validate the genotyping assays.

### Statistical analysis

All typed SNPs were evaluated for departure from Hardy-Weinberg equilibrium (HWE) using Haploview 4.2 (*P* > 0.001)^41^. TDT analysis was described in detail in a previous study^42^. Permutation testing was used to obtain a measure of significance corrected for multiple testing bias.

## Additional Information

**How to cite this article**: Zhao, S. *et al.* Family-based analysis of eight susceptibility loci in polycystic ovary syndrome. *Sci. Rep.* **5**, 12619; doi: 10.1038/srep12619 (2015).

## Supplementary Material

Supplementary Information

## Figures and Tables

**Table 1:** Clinical characteristics of 321 PCOS cases.

**Characteristics**	**Mean**	**SD**
Age (years)	27.06	3.72
BMI (kg/m^2^)	25.07	4.53
FSH (IU/L)	6.36	1.73
LH (IU/L)	11.27	6.56
Testosterone (ng/dl)	69.04	29.83

SD: standard deviation; BMI: body mass index; FSH: follicle stimulating hormone; LH: luteinizing hormone.

**Table 2:** TDT results from 321 PCOS family trios with 10 SNPs.

**SNP**	**CHR**	**Nearby Gene**	**Location**	**HWE *P***	**Over-T allele**	**T:U**	**TDTχ^2^**	***P***	**OR**	**PER *P***
rs2268361	2p16.3	*FSHR*	intron	0.881	G	137:131	0.134	0.71	1.05	1.00
**rs2349415**	**2p16.3**	***FSHR***	**intron**	**0.003**	**C**	**130:75**	**14.76**	**0.0001**	**1.73**	**0.0036**
rs4385527	9q22.32	*C9orf3*	intron	0.875	C	80:66	1.342	0.25	1.21	0.99
**rs3802457**	**9q22.32**	***C9orf3***	**intron**	**0.799**	**C**	**60:25**	**14.42**	**0.0001**	**2.4**	**0.0046**
rs1894116	11q22.1	*YAP1*	intron	0.802	T	98:93	0.131	0.72	1.05	1.00
rs705702	12q13.2	*RAB5B*	downstream	0.611	C	115:107	0.288	0.59	1.07	1.00
rs2272046	12q14.3	*HMGA2*	intron	0.855	T	50:31	4.457	0.035	1.61	0.62
rs4784165	16q12.1	*TOX3*	downstream	0.875	T	132:102	3.846	0.050	1.29	0.73
rs2059807	19p13.3	*INSR*	intron	0.863	T	124:107	1.251	0.26	1.16	0.99
rs6022786	20q13.2	*SUMO1P1*	downstream	0.064	G	149:124	2.289	0.13	1.20	0.96

CHR: chromosome; HWE: Hardy-Weinberg equilibrium; Over-T allele: over-transmitted allele; T:U: transmitted trios vs. un-transmitted trios; OR: odds ratio; PER: permutation; *P*: P value.
