# Supplementary material for: Family-based analysis of eight susceptibility loci in polycystic ovary syndrome
Source: Sci Rep. 2015 Jul 29;5:12619. doi: 10.1038/srep12619 (PMC4518258; doi:10.1038/srep12619)
Supplement: Supplementary Information [file srep12619-s1.pdf]

Supplementary Information

Family-based analysis of eight susceptibility loci in polycystic ovary syndrome

Shigang Zhao[a,b¶], Ye Tian[a¶], Xuan Gao[b¶], Xiuqing Zhang[b], Hongbin Liu[b,c], Li You[b], Yongzhi Cao[b], Shizhen Su[b], Wai-Yee Chan[c], Yun Sun[a], Han Zhao[b*], Zi-Jiang Chen[a,b*]

Supplementary Information: Titles and Captions

Supplementary Figure S1. The interaction networks of C9orf3 with other proteins predicated by String database. ACVR1B: activin A receptor, type IB; SLITRK1: SLIT and NTRK-like family, member 1; SUN2: unc-84 homolog B; DHFR: dihydrofolate reductase; ZP4: zona pellucida glycoprotein 4; INHBE: inhibin, beta E; AMZ1: archaelysin family metallopeptidase 1; LPA: lipoprotein, Lp (a); ATP7A: ATPase, Cu++ transporting, alpha polypeptide; FAM22F: family with sequence similarity 22, member F.

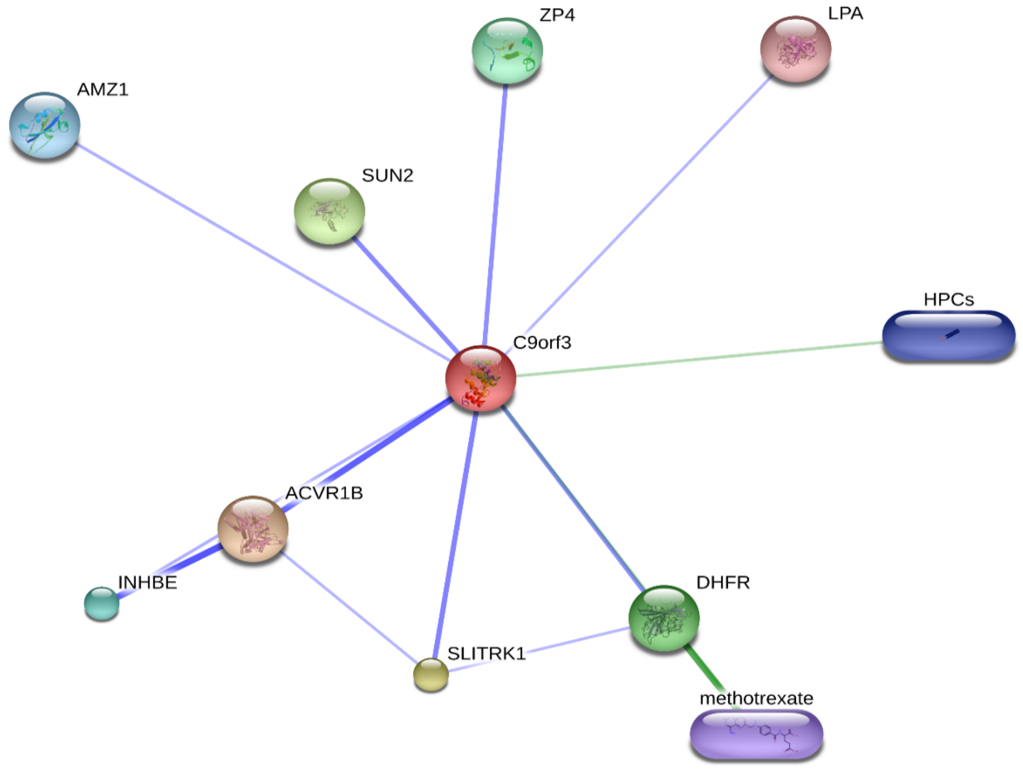

Supplementary Table S1. MAFs of 321 PCOS probands and 1510 PCOS GWAS subjects

SNP	CHR	Nearby Gene	MAF		
			GWAS-Control (n=2016)	GWAS-PCOS (n=1510)	TDT-PCOS (n=321)
rs2268361	2	*FSHR*	0.496	0.446	0.442
rs2349415	2	*FSHR*	0.181	0.227	0.189
rs4385527	9	*C9orf3*	0.219	0.148	0.148
rs3802457	9	*C9orf3*	0.096	0.060	0.071
rs1894116	11	*YAP1*	0.194	0.226	0.235
rs705702	12	*RAB5B*	0.245	0.304	0.321
rs2272046	12	*HMGA2*	0.093	0.066	0.077
rs4784165	16	*TOX3*	0.325	0.387	0.335
rs2059807	19	*INSR*	0.301	0.335	0.312
rs6022786	20	*SUMO1P1*	0.339	0.368	0.364

CHR: chromosome; MAF: Minor Allele Frequency.
